# Comprehensive Analysis of Bulk RNA‐Seq and Single‐Cell RNA‐Seq Data Unveils Sevoflurane‐Induced Neurotoxicity Through SLC7A11‐Associated Ferroptosis

**DOI:** 10.1111/jcmm.70307

**Published:** 2024-12-26

**Authors:** Xiaolan Hu, Yiping Zhang, Lian Guo, Renjie Xiao, Linhui Yuan, Fen Liu

**Affiliations:** ^1^ Department of Anesthesiology, the Second Affiliated Hospital, Jiangxi Medical College Nanchang University Nanchang China; ^2^ Department of Critical Care Medicine, the First Affiliated Hospital, Jiangxi Medical College Nanchang University Nanchang P. R. China; ^3^ Jiangxi Provincial Key Laboratory of Prevention and Treatment of Infectious Diseases, Jiangxi Medical Center for Critical Public Health Events, The First Affiliated Hospital, Jiangxi Medical College Nanchang University Nanchang Jiangxi P. R. China

**Keywords:** astrocyte, cognitive dysfunction, ferroptosis, neurotoxicity, sevoflurane

## Abstract

Sevoflurane's potential impact on cognitive function and neurodevelopment, especially in susceptible populations such as infants and the elderly, has raised widespread concern. This study focuses on how sevoflurane induces ferroptosis in astrocytes and identifies solute carrier family 7 member 11 (SLC7A11) as a mediator of ferroptosis, providing new insights into sevoflurane‐related neurotoxic pathways. We analysed single‐cell sequencing (scRNA‐seq) data from sevoflurane‐exposed mice and control mice, supplemented with bulk RNA‐seq data, to assess gene expression alterations. Additionally, pregnant mice were subjected to in vivo experiments, and in vitro studies using U251 astrocytoma cells were conducted to evaluate sevoflurane's neurotoxic effects on offspring, focusing on ferroptosis markers and SLC7A11 expression. Sevoflurane exposure led to learning, memory and behavioural deficits in offspring, associated with decreased SLC7A11 expression and increased signs of ferroptosis. In U251 cells, sevoflurane reduced cell viability, increased reactive oxygen species (ROS) levels and affected the expression of ferroptosis regulatory factors, supporting the hypothesis that sevoflurane induces astrocyte ferroptosis through SLC7A11 modulation. Molecular docking experiments suggest a direct interaction between sevoflurane and SLC7A11. This study provides mechanistic insights into sevoflurane‐induced neurotoxicity, emphasising the importance of SLC7A11 in regulating astrocyte ferroptosis. Our findings highlight the potential for targeting ferroptosis pathways to mitigate the adverse effects of sevoflurane anaesthesia.

**Abbreviations**
ACSL4: Acyl‐CoA synthetase long‐chain family member 4
CCK‐8: Cell counting Kit‐8
EdU: 5‐Ethynyl‐2′‐deoxyuridine
FPN1: Ferroportin 1
FTH1: Ferritin heavy chain 1
GFAP: Glial fibrillary acidic protein
GPX4: Glutathione peroxidase 4
HE: Haematoxylin and eosin
HO‐1: Haem oxygenase 1
IL‐1β: Interleukin 1 beta
IL‐6: Interleukin 6
MWM: Morris water maze
NRF2: Nuclear factor erythroid 2–related factor 2
PCA: Principal component analysis
ROS: Reactive oxygen species
SLC7A11: Solute carrier family 7 member 11
TFR1: Transferrin Receptor 1
TNF‐α: Tumour Necrosis Factor‐alpha

## 1 Introduction

Sevoflurane, a widely utilised volatile anaesthetic, presents a dichotomy in its effects on the brain, oscillating between neuroprotective benefits and potential neurotoxicity [1]. Studies highlight sevoflurane's neuroprotective roles, especially in mitigating cerebral ischaemia–reperfusion injury, reducing inflammation and offering protection in scenarios like subarachnoid haemorrhage and trauma [2, 3]. However, there is a growing concern about its neurotoxic impact, particularly in vulnerable populations such as infants and the elderly [4]. Evidence suggests sevoflurane could induce cognitive dysfunction, affect the gamma‐aminobutyric acidergic (GABAergic) system and trigger neuroinflammation, raising alarms over its developmental neurotoxicity and implications for long‐term neurocognitive outcomes [5, 6, 7, 8]. While the protective effects are undeniable in certain acute settings, the potential for adverse effects, especially with prolonged or early‐life exposure, cannot be overlooked, underscoring the complexity of sevoflurane's impact on the brain.

Recent studies employing single‐cell sequencing (scRNA‐seq) technologies have shed light on the nuanced impacts of exposure to sevoflurane, revealing alterations specific to different cell types within the brain that have significant implications for understanding sevoflurane‐induced neurotoxicity. Zhang et al. [9] demonstrated that sevoflurane affected synaptophysin and led to cognitive and motor deficits in young mice, with potential implications for paediatric anaesthesia practices. Chang et al.'s [10] work further elucidated the broad cellular impact of sevoflurane on human prefrontal cortex cells, indicating transient alterations in the transcriptome profiling across various cell types, including microglia and neurons. Song et al. [11] highlighted sevoflurane's affecting neuronal differentiation and microglia function differently in male and female mice. Finally, Zhao et al. [12] provided insight into prefrontal cortex cell‐type–specific changes postsevoflurane exposure, showing disruptions in the development of oligodendrocytes and glutamatergic neurons, which correlated with cognitive deficits. However, the aforementioned studies do not fully reveal the mechanisms of sevoflurane‐induced neurotoxicity and are incomplete. Reanalysing these single‐cell sequencing datasets and validating gene expression in animal and cellular models hold immense scientific value. It enables the identification of precise molecular pathways and cellular targets affected by sevoflurane, paving the way for developing targeted interventions to mitigate anaesthesia‐related neurodevelopmental risks.

Emerging evidence has highlighted the crucial role of ferroptosis, an iron‐dependent form of cell death [13], in mediating sevoflurane‐induced neurotoxicity. For instance, Wu et al. [14] and Cheng et al. [15] have established that sevoflurane disrupts iron metabolism in the brain, leading to iron overload and ferroptosis in neuronal cells. This process is mediated through specific signalling pathways, including the AMP‐activated protein kinase (AMPK)/mechanistic target of rapamycin (mTOR) pathway, and involves key proteins such as acyl‐CoA synthetase long‐chain family member 4 (ACSL4), which is fundamental in triggering the process of ferroptosis. Furthermore, the study conducted by Xu et al. [16] demonstrated that sevoflurane mediates ferroptosis in glioma cells via the activating transcription factor 4 (ATF4)–cation transporter HAC1 (CHAC1) signalling pathway. Despite these advances, the participation of astrocytes in ferroptosis induced by sevoflurane has not yet been documented in the literature. Investigating the role of ferroptosis in astrocytes after sevoflurane exposure could provide a comprehensive understanding of sevoflurane's impact on the brain and offer novel insights into mitigating its adverse effects.

## 2 Methods

### 2.1 Data Source

ScRNA‐seq dataset GSE139012 was retrieved from the Gene Expression Omnibus (GEO) database, accessible at https://www.ncbi.nlm.nih.gov/. This dataset comprises scRNA‐seq data from cortical samples of three mice treated with sevoflurane and three untreated control mice. Bulk RNA‐seq dataset GSE155770 containing hippocampus samples of four sevoflurane‐treated mice and four control mice was also downloaded from the GEO database.

### 2.2 ScRNA‐Seq Analysis

According to the European Nucleotide Archive (ENA) accession number: PRJNA578089, raw sequencing data for this project was obtained. We performed single‐cell data analysis using the CellRanger pipeline. Initially, we conducted quality control on the matrix file using the Seurat package [17] and filtered out cells with aberrant gene expression values (MADs = 3) using the scatter method. Data normalisation was carried out using ScaleData, and batch effect correction was performed using IntegrateData. After the quality control dataset, we selected the top 2000 genes for principal components analysis (PCA). Subsequently, we applied tSNE clustering with a resolution of 0.1 and used singleR for single‐cell annotation of the clustering results. The criteria for selecting differentially expressed genes (DEGs) were *p*‐value < 0.05, avg_log_2_FC > 0.1 or < −0.1. Functional annotation of DEGs was conducted using the Clusterprofiler package [18].

### 2.3 Bulk RNA‐Seq Analysis

Using R programming, an analysis of microarray data was conducted. First, the gene expression data from eight mouse tissue samples were imported. Quality control was performed based on the sample expression data, followed by PCA and differential expression analysis. Probe IDs were reannotated to obtain corresponding gene names, and functional annotation analysis was conducted for the DEGs.

### 2.4 Animal Ethics Statement

In the course of this study, the murine subjects were accommodated within specific pathogen‐free (SPF) animal housing conditions. These conditions maintained a constant ambient temperature of 24°C ± 1°C and relative humidity levels between 50% and 60%. Additionally, the facility provided a controlled photoperiod consisting of a 12‐h light/dark cycle. All housing and experimental protocols adhered strictly to the guidelines provided by the National Institutes of Health concerning the Care and Use of Laboratory Animals. Approval for the animal studies reported in this research was granted by the Laboratory Animal Welfare Ethics Committee at the First Affiliated Hospital of Nanchang University, under the approval number CDYFY‐IACUC‐202307QR005.

### 2.5 Sevoflurane Treatment

Female C57BL/6J mice, 7–8 weeks of age, were cohoused with male counterparts for breeding. Successfully mated female mice were housed individually. The morning after mating, vaginal smears were collected to confirm pregnancy by observing the presence of vaginal plugs.

On either Day 14 or 15.5 of gestation (referred to as G14 or G15.5), pregnant mice were systematically assigned to one of two distinct groups: The experimental group, which received sevoflurane treatment, and the control group, which did not receive this treatment. Sevoflurane anaesthesia was administered to pregnant mice during critical embryonic development stages, specifically from G14 to G16. In the sevoflurane group, the mice were subjected to a daily inhalation of 2.5% sevoflurane, which was administered in conjunction with 100% oxygen over a period of 2 h each day. In parallel, the control group mice inhaled 100% oxygen under identical conditions.

The sevoflurane anaesthesia procedure followed these steps: An anaesthetic chamber with dimensions of 20 × 20 × 10 cm^3^ was used. Anaesthesia induction was achieved with a flow rate of 2 L/min for 3 min, subsequently maintained at a rate of 1 L/min. Real‐time monitoring of anaesthesia and oxygen concentrations was conducted using a gas analyser (Ohmeda, Tewksbury, MA). The rectal temperature of the pregnant mice was strictly controlled to remain at 37°C ± 0.5°C. Following the administration of anaesthesia, the mice were exposed to an environment with 100% oxygen until the restoration of their righting reflex, which generally occurred within 20 min. Upon recovery from anaesthesia, the mice were carefully relocated to their original housing to recuperate.

At appropriate time points during postnatal development, specifically on postnatal Days 7 and 14, the offspring of the pregnant mice were selected for neurotoxicity assessments. These assessments included the observation of behavioural performance and the preparation of hippocampal tissue slices to evaluate neural cell survival, differentiation and migration. The pups are weaned at 21 days after birth and are subsequently used for biochemical studies (6 mice per group) and behavioural studies (6 mice per group).

### 2.6 Morris Water Maze Experiment

Before initiating the experiment, each mouse was isolated in a separate cage and introduced to a softly illuminated test room. The Morris water maze experiment was conducted in a circular pool with a diameter of 150 cm and a depth of 50 cm. This pool contained a 10 × 10 cm adjustable platform and was filled with harmless black ink, ensuring the surface of the water was 1 cm higher than the platform. The pool was segmented into four equal sections. Over the course of 5 consecutive days, the mice underwent a structured training regimen consisting of four trials per day. A trial was considered complete when a mouse located the platform or after 60 s had passed. The time taken and the route of each mouse to find the platform were documented, and their swimming speeds were measured. On the 6th day, the platform was withdrawn, and measurements were taken regarding the duration spent by the mice in the quadrant where the platform had previously been located, as well as the number of times they traversed the area of the former platform.

### 2.7 Open‐Field Test

An open‐field apparatus with dimensions of 50 × 50 × 50 cm was utilised for the open‐field test. The inner zone was defined as a square area located 10 cm away from the edges, while the remaining area was considered the outer zone. At the commencement of the experiment, each mouse was positioned within the inner zone of the apparatus. A video‐imaging apparatus provided by VJ Instruments was utilised to meticulously document the traversed distance by each subject and the duration of occupancy within the central zone. These measurements were systematically recorded over 10 min.

### 2.8 Novel Object Recognition

An open‐field apparatus with dimensions of 50 × 50 × 50 cm was utilised for the novel object recognition. In the open‐field test, two indistinguishable and nonscented objects were positioned equidistantly within the arena. Initially, the mice were introduced into the centre of the apparatus, maintaining an equal distance from each object. The video‐recording system was activated to record the mice's exploration time on each object. Subsequently, one previously utilised object was substituted with a novel item, following which the mice were identically reintroduced into the apparatus in an identical fashion. The exploration time for each object was recorded. The formula for calculating the recognition index (RI) is as follows:

$$\mathrm{RI}=\frac{\mathrm{New}\;\text{object}}{\mathrm{New}\;\text{object}+\mathrm{old}\;\text{object}}\times 100\%$$

### 2.9 Cell Culture

U251 astrocytoma cell lines were procured from the European Collection of Authenticated Cell Cultures (ECACC). These cells were maintained in Dulbecco's modified Eagle medium (DMEM) procured from Corning (Catalogue #10‐017‐CV; Corning, USA). The culture medium was supplemented with 10% foetal bovine serum (FBS, #A4766801; Gibco, USA) and 100 units/mL of penicillin–streptomycin (#15140122; Gibco, USA). The cells were maintained in the Forma Steri‐Cycle CO_2_ Incubator (#370; Thermo Fisher Scientific, USA) ensuring an environment with 5% CO_2_ at a constant temperature of 37°C.

Following treatment with various concentrations of SEV (0%, 1.2%, 2.4% and 3.6%) for specified durations (0, 3, 6 and 12 h), U251 astrocytoma cells were digested and collected. The gas flow rate was maintained at 1 L/min to ensure a consistent concentration of SEV, which was monitored using a multigas monitor (#S/5; Datex‐Ohmeda, Finland). To ensure accurate in vitro exposure to sevoflurane, we prepared the treatment solutions in a controlled environment, minimising evaporation. All treatments were conducted in a specialised chamber designed to contain volatile agents, maintaining stable concentrations of sevoflurane. Cells were exposed to the anaesthetic under these conditions, after which they were digested and collected for further analysis.

### 2.10 H&E Staining

The hippocampal tissues from differentially treated mice were processed for section preparation. These sections underwent deparaffinisation and rehydration before being immersed in haematoxylin dye (#H9627; Sigma‐Aldrich, USA). The sections were kept in this dye until the cell nuclei appeared blue, after which they were rinsed with distilled water. Subsequently, these slices were immersed in eosin dye (#15086‐94‐9; Sigma‐Aldrich, USA), removed when the cytoplasm displayed a pink hue and then rinsed again with distilled water. The sections were dehydrated and cleared once more before being sealed for observation.

### 2.11 Nissl Staining

After paraffin embedding, coronal sections (2.5 μm thick) were obtained from the hippocampal region. The sections were stained using a Nissl Staining Solution Kit (#N21480; Thermo Fisher Scientific, USA). Following dewaxing, the slices were immersed in cresyl violet solution and incubated in a constant temperature incubator at 60°C for 1 h. Poststaining, the sections were rinsed in PBS for 5 min. Differentiation of the slices was performed under a microscope. Images were collected from the CA1 region at 400× magnification, ensuring consistency in the sampled areas. The relative mean optical density values were calculated using ImageJ software for further quantitative analysis.

### 2.12 Immunohistochemistry

Following the processes of deparaffinisation, rehydration and antigen retrieval, the hippocampal tissue sections were treated to inhibit endogenous peroxidase activity using 3% hydrogen peroxide. Subsequently, nonspecific binding sites were blocked with 5% bovine serum albumin (BSA) to ensure specificity in subsequent immunostaining procedures. The tissue sections were subsequently incubated overnight at 4°C with the primary antibodies: anti–glial fibrillary acidic protein (GFAP) at a dilution of 1:400 (#PA1‐10004; Invitrogen, USA) and anti‐Ki67 at a dilution of 1:400 (#ab15580; Abcam, UK). The next day, after removing the unbound primary antibodies, the sections were treated with secondary antibodies, specifically goat anti‐rabbit IgG H&L (HRP) at a dilution of 1:200 (#ab97051; Abcam), and were maintained at room temperature for 1 h. Following DAB staining, haematoxylin counterstaining, hydrochloric acid alcohol differentiation, dehydration and sealing, the experimental results were observed and captured using the Leica DM4000B Fluorescent Microscope.

### 2.13 Immunofluorescence

U251 astrocytoma cells were seeded into a 96‐well plate at a density of 1 × 10^4^ cells per well. Following an incubation period of 12 h, the cells were subjected to treatment with various concentrations of sevoflurane (0%, 1.2%, 2.4% and 3.6%) for predetermined time intervals. For cell‐level immunofluorescence analysis, an antiferroportin 1 antibody (FPN1, 1:500; #PA5‐22993, Invitrogen, USA), antitransferrin receptor 1 antibody (TFR1, 1:500; #ab214039, Abcam, Cambridge, UK), antiferritin heavy‐chain 1 antibody (FTH1, 1:500, #ab75973; Abcam, Cambridge, UK), antinuclear factor erythroid 2‐related factor 2 antibody (NRF2, 1:500, #PA5‐27882, Invitrogen, USA) and antihaem oxygenase 1 antibody (HO‐1, 1:500, #ab189491; Abcam, Cambridge, UK) were utilised.

For tissue‐level immunofluorescence studies, hippocampal tissue sections from differentially treated mice were used. These sections were probed with an anti‐SLC7A11 antibody (1:500, #ab307601; Abcam, Cambridge, UK). The simplified procedure for tissue‐level immunofluorescence involves section preparation, blocking to prevent nonspecific binding, primary antibody incubation, secondary antibody incubation with a fluorophore‐conjugated antibody and finally, visualisation under Leica DM4000B Fluorescent Microscope.

### 2.14 Elisa

Mouse interleukin‐1 beta (IL‐1β) ELISA kit (#MLB00C; R&D Systems, USA), mouse interleukin‐6 (IL‐6) ELISA kit (#M6000B; R&D Systems, USA) and mouse tumour necrosis factor‐alpha (TNF‐α) ELISA kit (#MTA00B; R&D Systems, USA) were used to detect the concentrations of inflammatory cytokines. Samples from mice hippocampal tissue or cell culture media were collected. Microtiter plates were precoated with capture antibodies. Unbound capture antibodies were removed and blocked with 5% skim milk. Samples or standards for incubation were added. Following incubation, detection antibodies were added and removed and HRP‐conjugated secondary antibodies were added for incubation. Subsequently, TMB substrate was added. The OD_450_ nm values of each well were measured by using a Thermo Fisher Scientific ELISA reader (#1425955; Thermo Fisher, USA) and the concentrations of each inflammatory factor were calculated based on the standard curves.

### 2.15 qRT‐PCR

Total mRNA was isolated from the hippocampal tissues and cells of mice employing the Trizol Reagent (#15596018; Invitrogen, USA). Subsequently, cDNA synthesis was conducted using the SuperScript IV First‐Strand Synthesis System (#18091050; Thermo Fisher Scientific, USA). Then, qRT‐PCR analysis was performed with SYBR Premix ExTaq II (#50‐444‐027, Thermo Fisher Scientific, USA). $2^{-△△C_{T}}$ method was used to determine the relative mRNA expression with β‐actin as the reference gene. The PCR primers used in this study are listed in Table S1.

### 2.16 Western Blot

Proteins were extracted from mice hippocampal tissue and cells and their concentrations were determined via BCA assay. Protein extracts were first resolved by SDS‐PAGE and subsequently transferred onto PVDF membranes. Following this, the membranes were blocked using a 5% solution of skim milk, after which they were incubated with primary antibodies at 4°C overnight. The primary antibodies used were as follows: anti‐SLC7A11 antibody (1:1000, #ab307601; Abcam, Cambridge, UK), anti‐FPN1 antibody (1:1000, #PA5‐22993; Invitrogen, USA), anti‐TFR1 antibody (1:1000, #ab214039; Abcam, Cambridge, UK), anti‐FTH1 antibody (1:1000, #ab75973; Abcam, Cambridge, UK), anti‐antibody NRF2 (1:1000, #PA5‐27882; Invitrogen, USA), anti‐HO‐1 antibody (1:2000, #ab189491; Abcam, Cambridge, UK), anti‐ACSL4 antibody (1:1000, #22223‐1‐AP; Proteintech, Chicago, USA), anti‐GPX4 antibody (1:1000, #ab125066; Abcam, Cambridge, UK), anti‐cleaved‐caspase3 antibody (1:1000, #25128‐1‐AP; Proteintech, Chicago, USA), anti‐cleaved‐caspase7 antibody (1:1000, #9491; Cell Signalling Technology, Danvers, USA), anti‐cleaved‐PARP1 antibody (1:1000, #ab32064; Abcam, Cambridge, UK) and anti‐β‐actin antibody (1:1000, #ab213262; Abcam, Cambridge, UK). The next day, the membranes were incubated with secondary antibodies, goat anti‐rabbit IgG H&L (HRP) (1:2000, #ab97051; Abcam, Cambridge, UK), at room temperature for 2 h. Finally, DAB staining was performed and a chemiluminescence imaging system ChemiDoc MP imager (Bio‐Rad, Hercules, California, USA) was used for imaging.

### 2.17 CCK‐8 Assay

U251 astrocytoma cells were plated in a 96‐well plate at a density of 10,000 cells per well. Twelve hours postseeding, the cells were exposed to incremental concentrations of sevoflurane (0%, 1.2%, 2.4% and 3.6%) over specific durations (0, 3, 6 and 12 h). Subsequently, the original medium was replaced with the fresh medium containing the cell viability reagent CCK‐8 (#88–7485‐86; Thermo Fisher Scientific, United States) and incubated until the colour turned orange. The absorbance values at 450 nm of each well were measured using a Microplate Reader (BioTek).

U251 astrocytoma cells were inoculated in a 96‐well format at a density of 1 × 10^4^ cells per well, and were treated with 0% and 3.6% sevoflurane for 6 h, followed by treatment with ferrostatin‐1 (2 μM). The medium was then replaced with diluted CCK8 reagent, and the absorbance at 450 nm was quantified using a microplate reader following incubation periods of 1, 2, 3 and 4 days.

### 2.18 Reactive Oxygen Species (ROS) Detection

Intracellular ROS levels were quantified using the Highly Sensitive DCFH‐DA ROS Assay Kit (Catalogue #R252; DOJINDO, Japan). U251 astrocytoma cells were exposed to incremental concentrations of sevoflurane (0%, 1.2%, 2.4% and 3.6%) for specified durations (0, 3, 6 and 12 h). Subsequently, cells were enzymatically dissociated and harvested for analysis. The collected cells were coincubated with prediluted DCFH‐DA dye in an incubator for 20 min. To eliminate any unbound DCFH‐DA dye, the cells were rinsed thoroughly with PBS. Subsequently, flow cytometry was employed to accurately measure the DCF fluorescence intensity within each cell.

U251 cells were treated with sevoflurane (0%, 3.6%) for 6 h, followed by subsequent treatment with 2 μM ferrostatin‐1. The cells harvested were subjected to incubation with DCFH‐DA dye, already diluted, for a duration of 20 min. Subsequently, the DCF fluorescence intensity within each cell was quantitatively assessed using flow cytometry.

### 2.19 EdU

U251 astrocytoma cells were plated at a density of 10,000 cells per well in a 96‐well format. Following an incubation period of 12 h, the cells were exposed to incremental concentrations of sevoflurane (0%, 1.2%, 2.4% and 3.6%) for the indicated time. The diluted EdU reagent (#C10338, Ribobio, Guangzhou, China) was added and incubated with the cells for 2 h according to the manufacturer’s instructions. Following incubation, the medium containing EdU was discarded, and the cells were fixed with 4% paraformaldehyde. Subsequently, the click reaction was performed, and the cell nuclei were stained with DAPI. The experimental results were observed using a fluorescent microscope.

In a 96‐well plate, U251 cells were inoculated and treated with sevoflurane (0% and 3.6%) for 6 h, followed by further treatment with 2 μM ferrostatin‐1. The proliferation levels of cells in each group were examined by using a fluorescence microscope.

### 2.20 Flow Cytometry Detects Apoptosis

Following treatment with various concentrations of sevoflurane (0%, 1.2%, 2.4% and 3.6%) for specified durations (0, 3, 6 and 12 h), U251 astrocytoma cells were digested and collected. The cells were then incubated with Annexin V‐FITC binding solution and PI (2 μg/mL) for 20 min at 4°C. Fluorescence of each cell was subsequently analysed using flow cytometry (BD FACS Calibur, BD Biosciences).

U251 cells were treated with sevoflurane (0%, 3.6%) for 6 h, followed by subsequent treatment with 2 μM ferrostatin‐1. The cells from each group were collected and incubated with Annexin V‐FITC binding solution and PI at 4°C for 20 min. The fluorescence intensity of each cell was subsequently quantified by using a flow cytometer.

### 2.21 Molecular Docking Experiments

We retrieved the three‐dimensional (3D) structure of sevoflurane from the PubChem database (https://pubchem.ncbi.nlm.nih.gov/). Subsequently, we employed the OpenBabel software to convert the Structure‐Data File (SDF) format to a mol2 file format for further analysis. The amino acid sequences of solute carrier family 7 member 11 (SLC7A11) were downloaded from the NCBI database, the structure prediction was constructed from the sequence de novo using alphaFold2 software, the parameters were defaulted to alphaFold2 software and the results of Top 1 in the structure construction results were used as the 3D structures of SLC7A11 proteins used in this analysis. We then used the LeDock software to molecularly dock SLC7A11 and sevoflurane, with the parameters set to the LeDock software default. From the docking results, we chose the conformation with the lowest binding energy (the most stable) as the docking junction of SLC7A11 and sevoflurane molecules obtained from this analysis and the stable docking results were analysed and visualised using Pymol software.

### 2.22 Statistical Analysis

Statistical analyses were conducted utilising SPSS software (SPSS Inc., Chicago, IL) and GraphPad Prism 9.5.1 (GraphPad Software, San Diego, CA). Differences between groups were evaluated using Student's *t*‐tests. All data were presented as means ± standard deviation (SD). *p* < 0.05 was considered statistically significant.

## 3 Results

### 3.1 scRNA‐Seq Analysis

**FIGURE 1:**
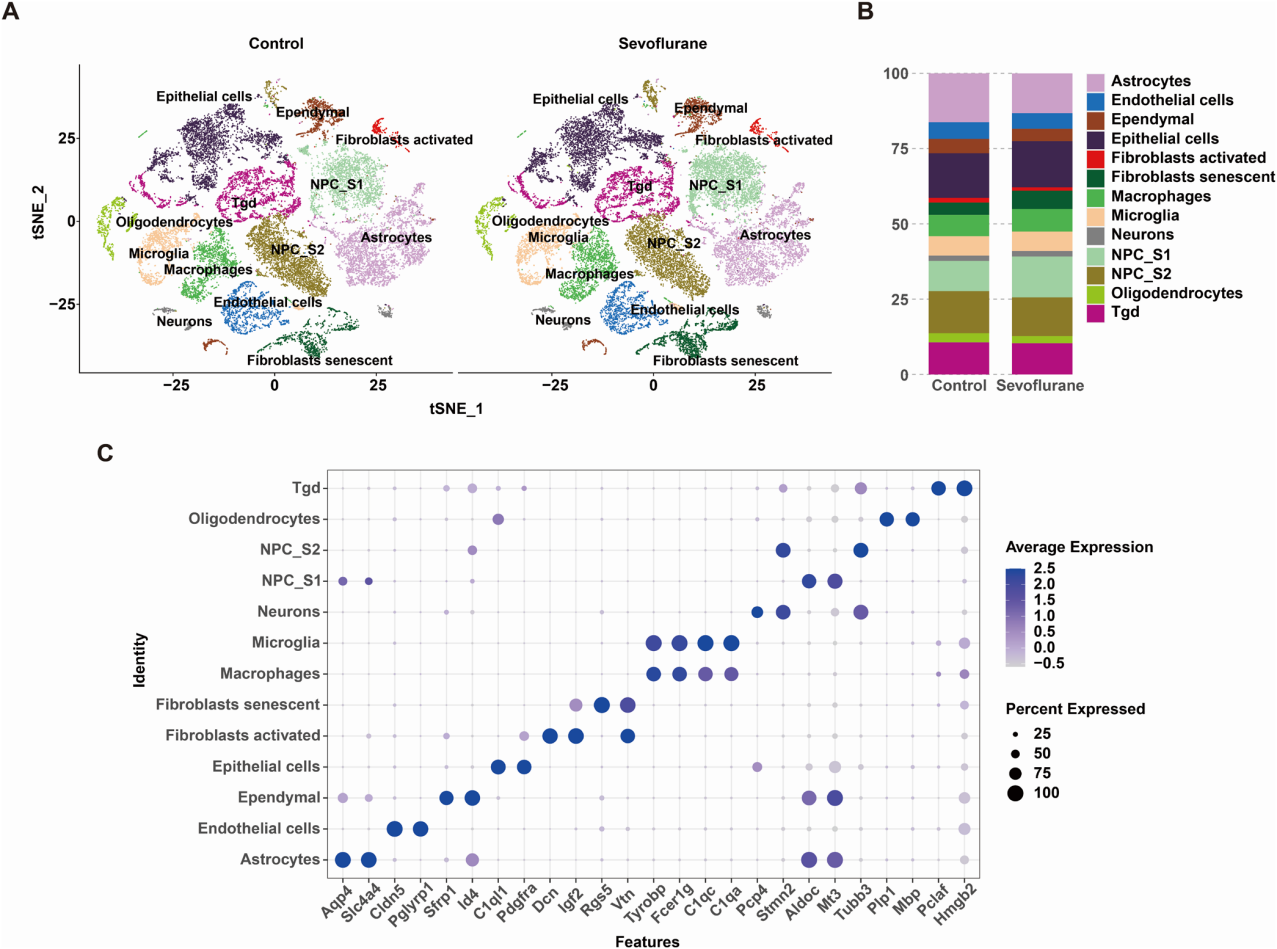
ScRNA‐seq analysis. (A) Cell annotation identified distinct cell types in the cortex samples from both control and sevoflurane‐treated groups. (B) Biomarkers expression of different cell types. (C) Proportions of different cell types in control and sevoflurane‐treated cortex samples.

We first analysed the scRNA‐seq data from three sevoflurane‐treated mice and three control mice in the GSE139012 dataset. After quality control and normalisation, we selected the Top 2000 genes for tSNE clustering analysis. Based on gene expression data, we used SingleR to annotate cell types for various cell populations. As shown in Figure 1A, we annotated 13 cell subtypes, including two distinct subgroups of neural progenitor cells (NPC), NPC‐S1 and NPC‐S2, with their biomarkers being Aldoc/mt3 and Stmn2/Tubb3, respectively (Figure 1B). Figure 1C and Table S2 show the proportions of various cell subtypes in these two sample groups. In the sevoflurane group, the proportion of astrocytes in mouse hippocampal tissue was lower (13.21%) compared to the control group (16.26%), suggesting that sevoflurane treatment may lead to astrocyte damage or death. We also observed an increase in the proportion of NPC_S1 (13.54%) and a decline in NPC_S2 in the sevoflurane group, indicating that sevoflurane may regulate NPC differentiation processes in the cerebral cortex.

### 3.2 Gene Function and Pathway Enrichment Analysis of DEGs in Astrocytes and Microglia

**FIGURE 2:**
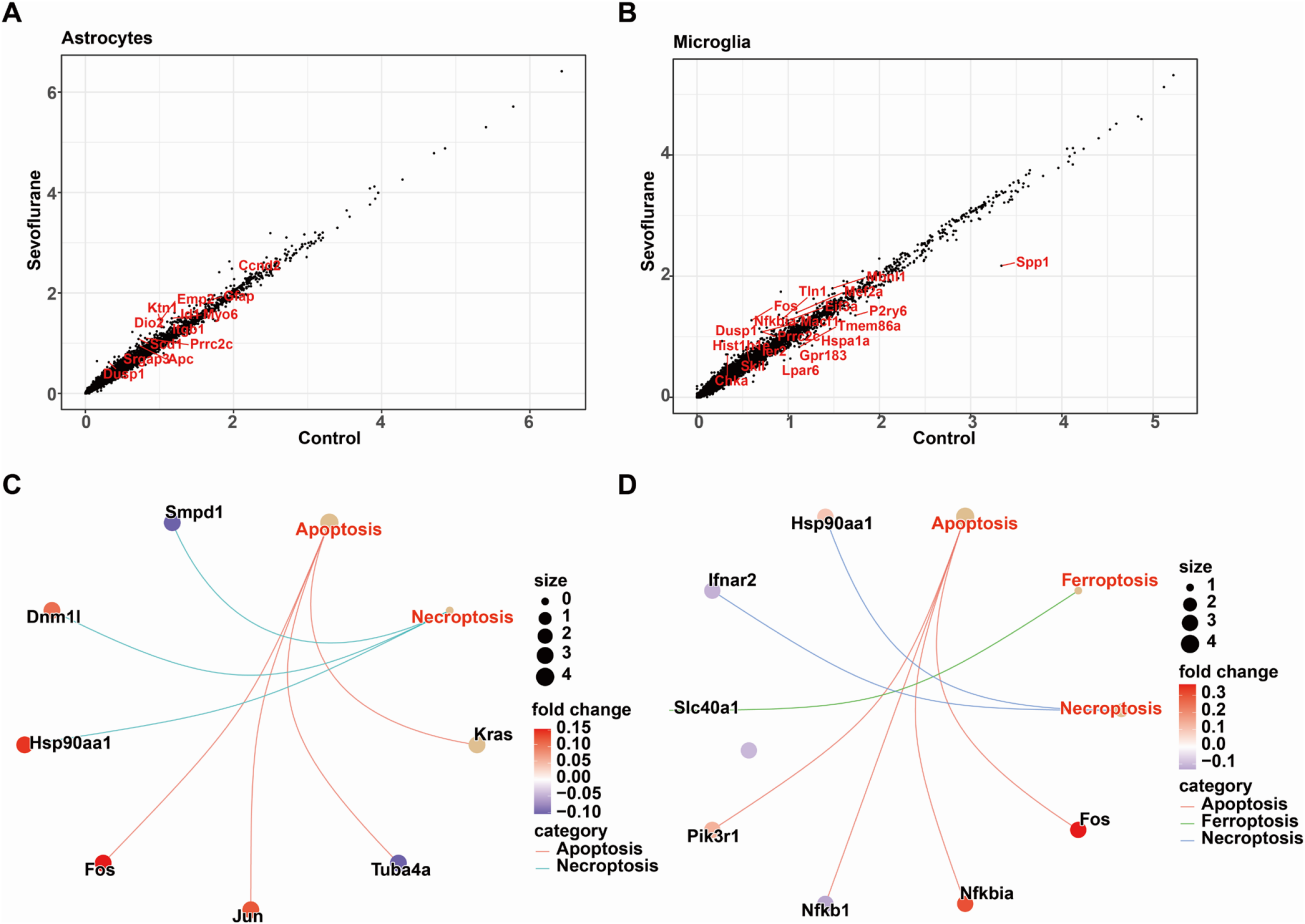
Gene function and signal pathway enrichment analysis of DEGs in astrocytes and microglia. Differentially expressed genes were identified in (A) astrocytes and (B) microglia. Expression pattern of genes involved in apoptosis, ferroptosis and necroptosis in (C) astrocytes and (D) microglia.

Astrocytes and microglia are two major types of glial cells in the nervous system. In sevoflurane‐treated mice, the proportion of astrocytes in the cerebral cortex decreased, however, the microglial composition did not show a significant difference between the two groups (Table S2). DEGs from astrocytes and microglia were identified separately, with the screening criteria being *p*‐value < 0.05 and avg_log_2_FC > 0.1 or < −0.1 (Figure 2A,B).

Functional analysis of DEGs in astrocytes revealed that, in terms of cell component, DEGs were mainly enriched in postsynaptic density, asymmetric synapse, neuron‐to‐ neuron synapse, as these are the components involved in the supporting and separating of neurons by astrocytes in the nervous system. At the biological process (BP) level, DEGs were mainly associated with axonogenesis, chromatin organisation and regulation of neurogenesis and processes related to neuronal proliferation and regulation. In terms of molecular function (MF), DEGs were predominantly associated with enrichment in tubulin binding, actin binding and microtubule binding, which may be related to astrocyte cell division and the formation of glial scars following central nervous system damage (Figure S1A). In the Kyoto Encyclopedia of Genes and Genomes (KEGG) pathways, DEGs were enriched in the Axon guidance pathway, indicating that sevoflurane may affect axon growth and guidance processes, thereby influencing neuronal development and function (Figure S1B).

Functional analysis of microglia revealed that DEGs were primarily enriched in functions related to mRNA processing, regulation of translation, RNA splicing, mRNA binding, GTPase binding and ubiquitin protein ligase binding, indicating rapid activation of microglia with transcription and translation of related genes (Figure S1C). In addition, KEGG pathways enrichment results indicated the involvement of DEGs in pathways related to inflammation and infection, such as human cytomegalovirus/Salmonella/Yersinia infection, as well as immune responses, including the T cell receptor signalling pathway and Toll‐like receptor signalling pathway, suggesting that sevoflurane treatment may trigger an immune‐inflammatory response in the nervous system (Figure S1D).

It is noteworthy that genes related to apoptosis and necroptosis exhibited expression changes in astrocytes, with genes like Jun, Fos and Dnm1l being upregulated, while genes like Tuba4a were downregulated (Figure 2C). In microglia, apoptosis‐related genes were also highly expressed, possibly due to their involvement in mediating the inflammatory response (Figure 2D).

### 3.3 Different Subtypes of NPC

**FIGURE 3:**
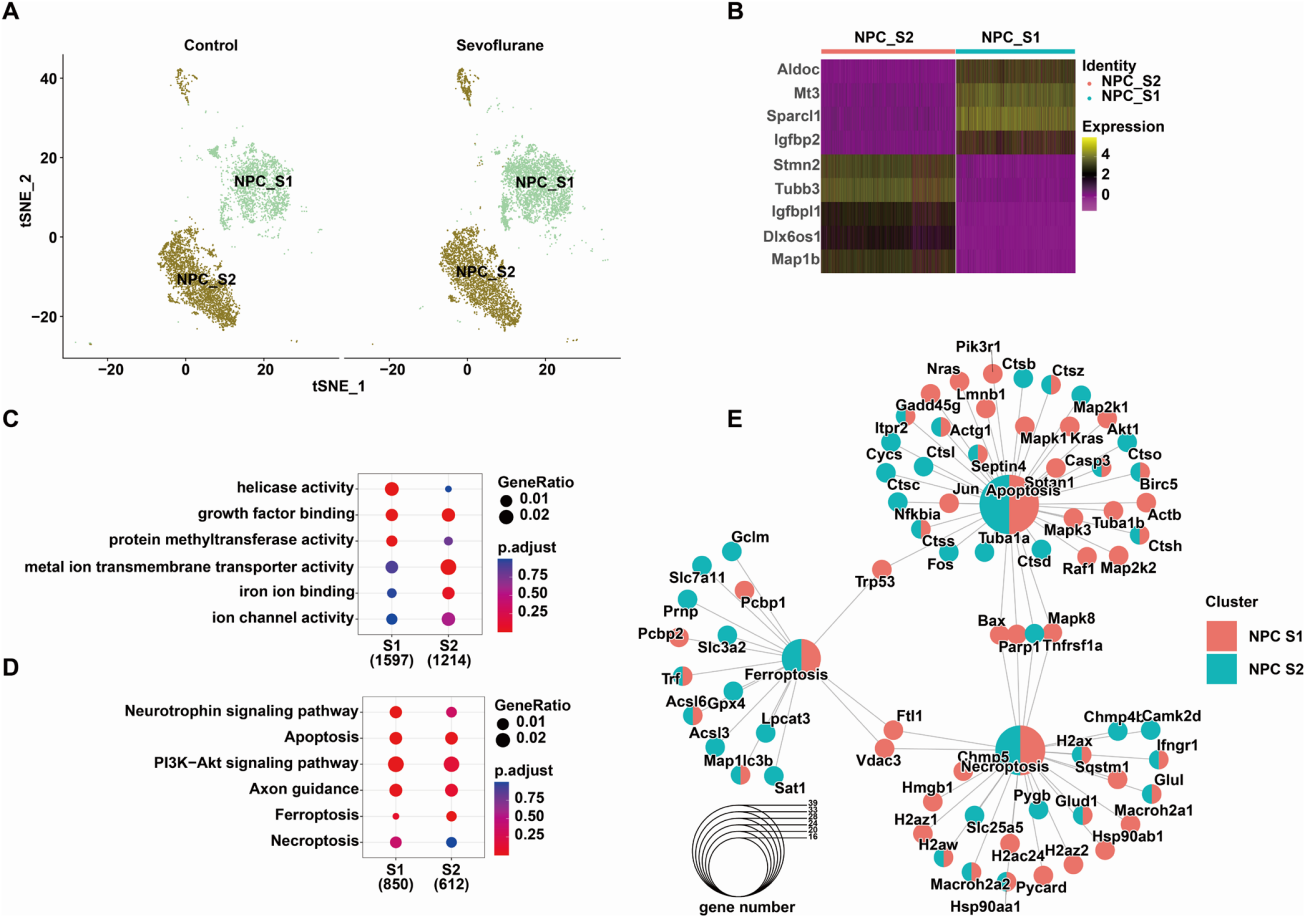
Different subtypes of neural progenitor cells. (A) There were two subtypes identified in neural progenitor cells, namely, NPC‐S1 and NPC‐S2. (B) Expression patterns, (C) BP analysis, (D) KEGG pathway analysis of marker genes in NPC‐S1 and NPC‐S2 and (E) pathway interaction network.

NPC are undifferentiated cells characterised by their pluripotency or multipotency, capable of self‐renewal and differentiation into various cell types, including neurons and neuroglial cells, playing a significant role in neurodevelopment, neural injury and repair. Cell annotations showed that there were two subtypes of NPC in the cerebral cortex, namely, NPC‐S1 and NPC‐S2 subtypes (Figure 3A). They exhibited distinct expression patterns of marker genes (Figure 3B), and sevoflurane could induce changes in their proportions: NPC‐S1 subtype increased in the sevoflurane‐treated group, while NPC‐S2 subtype decreased in the treated group. Combining marker gene expression patterns in the two subgroups, we performed functional annotations. As shown in Figure 3C, compared to the NPC S1 subtype, genes in the NPC S2 subtype were primarily involved in iron ion binding, ion channel activity and metal ion transmembrane transporter activity, suggesting that the S2 subgroup may be associated with metal ion binding and transport. KEGG pathway analysis indicated that both subgroups showed enrichment in the phosphatidyl‐inositol 3‐kinase/serine–threonine kinase (PI3K‐Akt) signalling pathway and Axon guidance, which may be associated with their proliferation and differentiation capacities. In addition, in the S2 subtype, we observed the enrichment of pathways related to ferroptosis, apoptosis and necroptosis (Figure 3D). Furthermore, visualising the interactions between pathways, we found that both S1 and S2 subtypes enriched in genes related to ferroptosis, apoptosis and necroptosis (Figure 3E), indicating that sevoflurane may regulate NPC death to cause neural system damage, and the forms of death may differ between the different subtypes.

### 3.4 Coanalysis of Bulk RNA‐Seq and scRNA‐Seq

**FIGURE 4:**
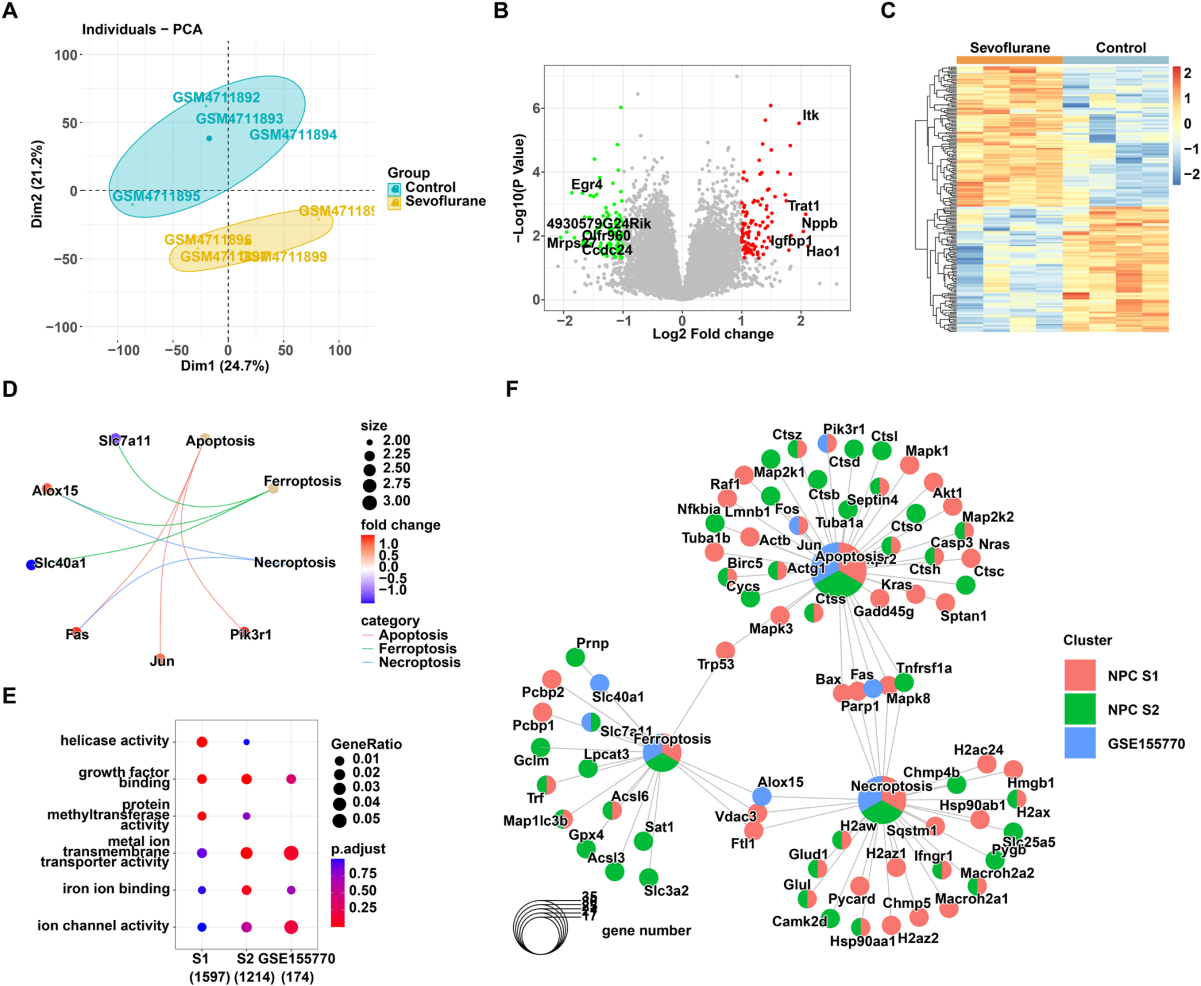
Coanalysis of bulk RNA‐seq and scRNA‐seq. (A) PCA analysis of the GSE155770 dataset. (B) Differentially expressed genes were identified in GSE155770 with *p* < 0.05 and Log_2_Fold change > 1 or < −1. (C) Heatmap of DEGs. (D) Expression pattern of genes involved in apoptosis, ferroptosis and necroptosis. (E) GO analysis of NCP subtypes and GSE155770 dataset. (F) Pathway interaction network.

Bulk RNA‐seq data were analysed to validate the result of scRNA‐seq analysis. No significant outlying gene expression data were observed in control and sevoflurane‐treated cortex samples after PCA analysis (Figure 4A). Differential expression analysis was conducted using limma under *p* < 0.05 and Log_2_Fold change > 1 or < −1 (Figure 4B). As shown in Figure 4C, the control and sevoflurane‐treated cortex exhibited distinct gene expression patterns. Functional analysis revealed that, in terms of BP and MF, DEGs predominantly participate in pathways associated with metal ions, including their response, export and transmembrane transport activities, suggesting that neurological damage induced by sevoflurane may be associated with iron‐related cell death (Figure S1E). On the other hand, KEGG pathway analysis indicated enrichment of inflammatory responses and pathways related to iron death, such as hepatitis B/C, viral carcinogenesis and ferroptosis, implying that sevoflurane treatment in mice led to brain neural injury, triggering inflammation and related cell iron death processes (Figure S1F). Further analysis of cell death pathways revealed significant changes in genes associated with apoptosis, ferroptosis and necroptosis. Genes like Alox15, Pik3r1 and Fas showed upregulation in the sevoflurane‐treated group, while genes like SLC7A11 were significantly downregulated, with statistical significance (Figure 4D). The outcomes align with the observations derived from the scRNA‐seq analysis.

Furthermore, joint functional enrichment analysis indicated that the NPC‐S2 subtype showed highly consistent gene ontology (GO) analysis results with GSE155770. As depicted in Figure 4E, the marker genes associated with the NPC‐S2 subtype and the DEGs identified in the dataset GSE155770 predominantly relate to functionalities involving transmembrane transporter activity for metal ions and binding of iron ions, or ion channel activity, suggesting their potential involvement in iron‐related cell death. Functional network analysis demonstrated that these genes were predominantly associated with the ferroptosis and apoptosis pathways (Figure 4F). This confirmed the accuracy of our scRNA‐seq analysis, indicating that sevoflurane may affect hippocampal tissue cells, including NPC S2 or other neural cells, through iron death or apoptosis, contributing to the brain injury process.

### 3.5 Sevoflurane Treatment Induced Neurotoxicity and Downexpression of SLC7A11

**FIGURE 5:**
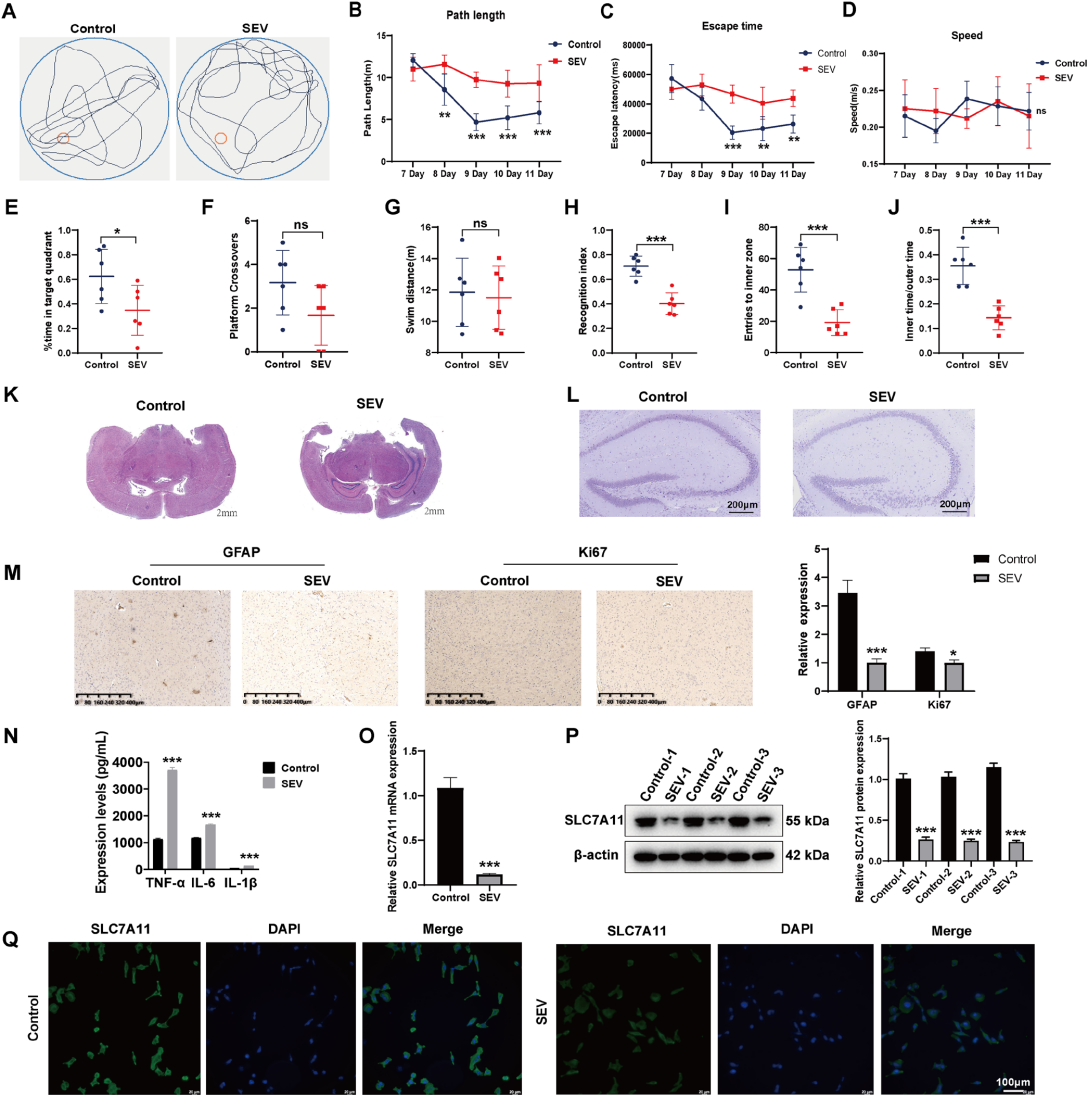
Sevoflurane treatment induced neurotoxicity and downexpression of SLC7A11. (A) Representative route of mice, (B) path length, (C) escape time, (D) speed, (E) time spent in the quadrant where the former escape platform was located, *p* = 0.0475, (F) the number of times crossing the platform, *p* = 0.0973, and (G) swim distance of offspring born from female mice receiving different treatments, *p* = 0.7849. (H) Recognition index, *p* < 0.001, (I) the frequency of entry into the inner region, *p* < 0.001, and (J) the percentage of time spent in the inner zone (inner time) compared to the time spent in the outer zone (outer time) of offspring born from female mice receiving different treatments, *p* < 0.001. Relative (K) H&E staining. (L) Nissl staining (scar bar = 200 μm) and (M) immunohistochemistry images of mice hippocampal tissue. (N) Expression levels of inflammation factors in mice brain tissue. (O) mRNA and (P) protein expression levels of SLC7A11 in mice hippocampal tissue. (Q) Cellular location of SLC7A11, scar bar = 100 μm. ‘ns’ indicates nonsignificant, 'ns' *p* ≥ 0.05; *p < 0.05; **p < 0.01; ***p < 0.001.

Astrocytes, the largest type of glial cells, are essential for maintaining the stability and function of the central nervous system. We, therefore, focused on SLC7A11 to specifically investigate the side effects of sevoflurane on astrocytes. To construct an animal model, C57BL/6J pregnant mice were subjected to sevoflurane or control treatment and the offspring of these mice underwent neurotoxicity assessments. In the Morris water maze experiment, although swimming speed was similar, the offspring of mice exposed to sevoflurane exhibited prolonged navigation times and paths to locate the escape platform, in contrast to the control counterparts (Figure 5A–D). Following the removal of the escape platform, they exhibited a reduced duration of presence and lower frequency of entries into the quadrant that previously contained the platform compared to the control group (Figure 5E–G). In novel object recognition and open‐field experiments, these offspring exhibited a decreased ability to detect new objects and explore new environments (Figure 5H–J). These results mentioned above indicated that the use of sevoflurane treatment in pregnant mice led to learning and memory impairments, decreased exploratory behaviour and increased anxiety towards new environments in the offspring. The results of HE staining and Nissl staining showed that, compared to the intact neurons and normal cell arrangement in the dentate gyrus (DG) region of the control group mice, the hippocampal tissues in the DG region of sevoflurane‐treated mice exhibited pathological changes such as cell oedema, degeneration, necrosis and significant disorganisation of cell structure (Figure 5K). Additionally, there was a decrease in the number of Nissl bodies, deformation in their morphology and weakened staining intensity, indicating severe damage to hippocampal neurons (Figure 5L).

Additionally, the levels of GFAP and Ki67 in the hippocampal tissues of these mice were lower than those in the control group (Figure 5M), which may suggest that the intervention with sevoflurane significantly inhibited astrocyte proliferation and activation. The upregulation of TNF‐α, IL‐6 and IL‐1β levels in sevoflurane‐treated mice also indicates the occurrence of inflammation in the mice (Figure 5N). These together validated that sevoflurane anaesthesia in pregnant mice induced neurotoxicity in foetal mice. Expression level and location of SLC7A11 were next detected to explore if sevoflurane treatment led to the dysregulation of SLC7A11. The mRNA and protein levels of SLC7A11 were both downregulated (Figure 5O,P); however, its location was not affected (Figure 5Q). Since SLC7A11 inhibits ferroptosis by regulating the activity of glutathione peroxidase, we hypothesise that the downregulation of SLC7A11 induced by sevoflurane may lead to the occurrence of ferroptosis.

### 3.6 Sevoflurane Caused U251 Astrocyte Ferroptosis by Regulating SLC7A11

**FIGURE 6:**
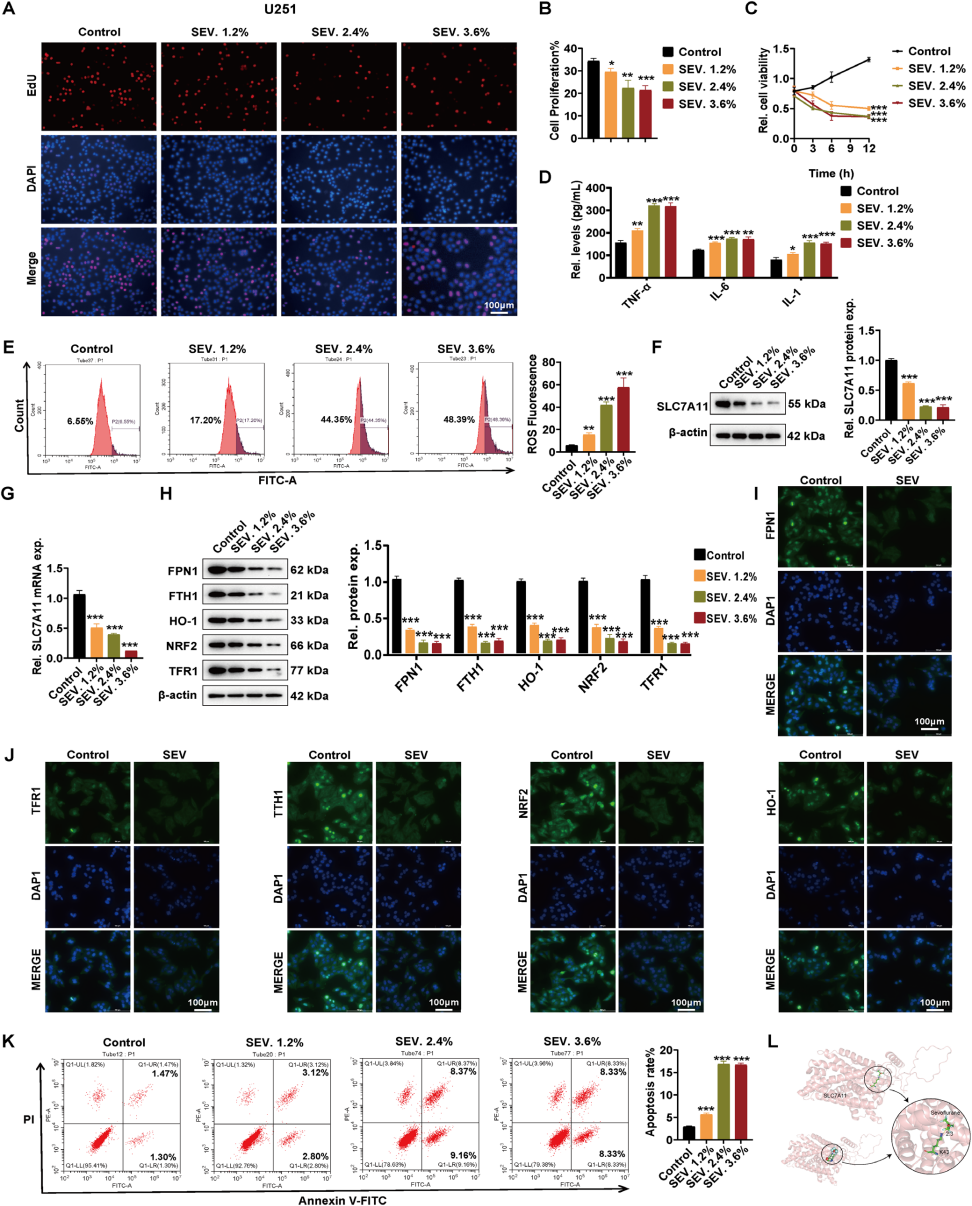
Sevoflurane caused U251 astrocyte ferroptosis by regulating SLC7A11. (A) EdU assay to detect cell proliferation capacity. (B) Statistical analysis of (A). (C) Utilisation of the CCK‐8 assay to evaluate cellular viability following exposure to sevoflurane. (D) Inflammation factors levels in the medium. (E) ROS levels in U251 astrocyte after sevoflurane treatment. (F) Protein and (G) mRNA levels of SLC7A11 in U251 astrocyte after sevoflurane treatment. (H) Expression levels of FPN1, TFR1, FTH1, NRF2 and HO‐1 in U251 astrocyte after sevoflurane treatment. (I, J) Immunofluorescence of FPN1, TFR1, FTH1, NRF2 and HO‐1 in U251 cells after 2.4% sevoflurane treatment. (K) The apoptosis rate of U251 astrocyte after sevoflurane treatment. (L) Schematic diagram of the molecular docking of SLC7A11 and sevoflurane. Scar bar = 100 μm. *n* = 3 biologically independent samples. *p < 0.05; **p < 0.01; ***p < 0.001.

We chose U251 astrocyte to investigate the regulated role of SLC7A11 on ferroptosis. U251 astrocytes were first treated with varying concentrations of sevoflurane, and 0% sevoflurane was set as the control group. As shown in Figure 6A–C, sevoflurane exposure led to a reduction in U251 astrocyte viability in a manner dependent on both concentration and exposure duration. Notably, concentrations of 2.4% and 3.6% sevoflurane exhibited comparable effects on the cells. We also detected relatively high levels of inflammation factors in the medium (Figure 6D), which was consistent with in vivo experiments. Next, we detected the ferroptosis status in U251 astrocytes. Sevoflurane led to an increase in ROS levels (Figure 6E), a key factor contributing to ferroptosis. Interestingly, sevoflurane‐treated U251 astrocytes exhibited lower mRNA and protein levels of SLC7A11 compared to the control group (Figure 6F,G), along with decreased expression of other ferroptosis‐related genes, including FPN1, FTH1, TFR1, NRF2 and HO‐1 (Figure 6H). Additionally, we examined the subcellular localisation and expression of these proteins using immunofluorescence assays and found that their expression levels decreased after treatment with 2.4% sevoflurane, but their subcellular localisation remained unchanged (Figure 6I,J). On the other hand, we also observed that in addition to ferroptosis, sevoflurane induced apoptosis in astrocytes (Figure 6K).

To further explore the mechanism by which sevoflurane affects SLC7A11, we conducted molecular docking experiments to simulate the interaction between sevoflurane and SLC7A11(Figure 6L). Through molecular docking analysis, we found that sevoflurane forms a stable hydrogen bond with the K43 residue of SLC7A11, with a hydrogen bond distance of 2.3 Å, indicating that sevoflurane can directly bind to this site. Additionally, due to the absence of a benzene ring in the sevoflurane molecule, no π‐π interactions were observed. Nevertheless, sevoflurane's binding via hydrogen bonding may alter the conformation or activity of SLC7A11, thereby affecting its function. These findings suggest that sevoflurane may directly regulate key sites of SLC7A11, further participating in the regulation of astrocyte ferroptosis and highlighting its potential role in neurotoxicity mechanisms.

### 3.7 Ferrostatin‐1 Ameliorates Sevoflurane‐Induced Downregulation of SLC7A11 Expression Leading to Ferroptosis in Astrocytes

**FIGURE 7:**
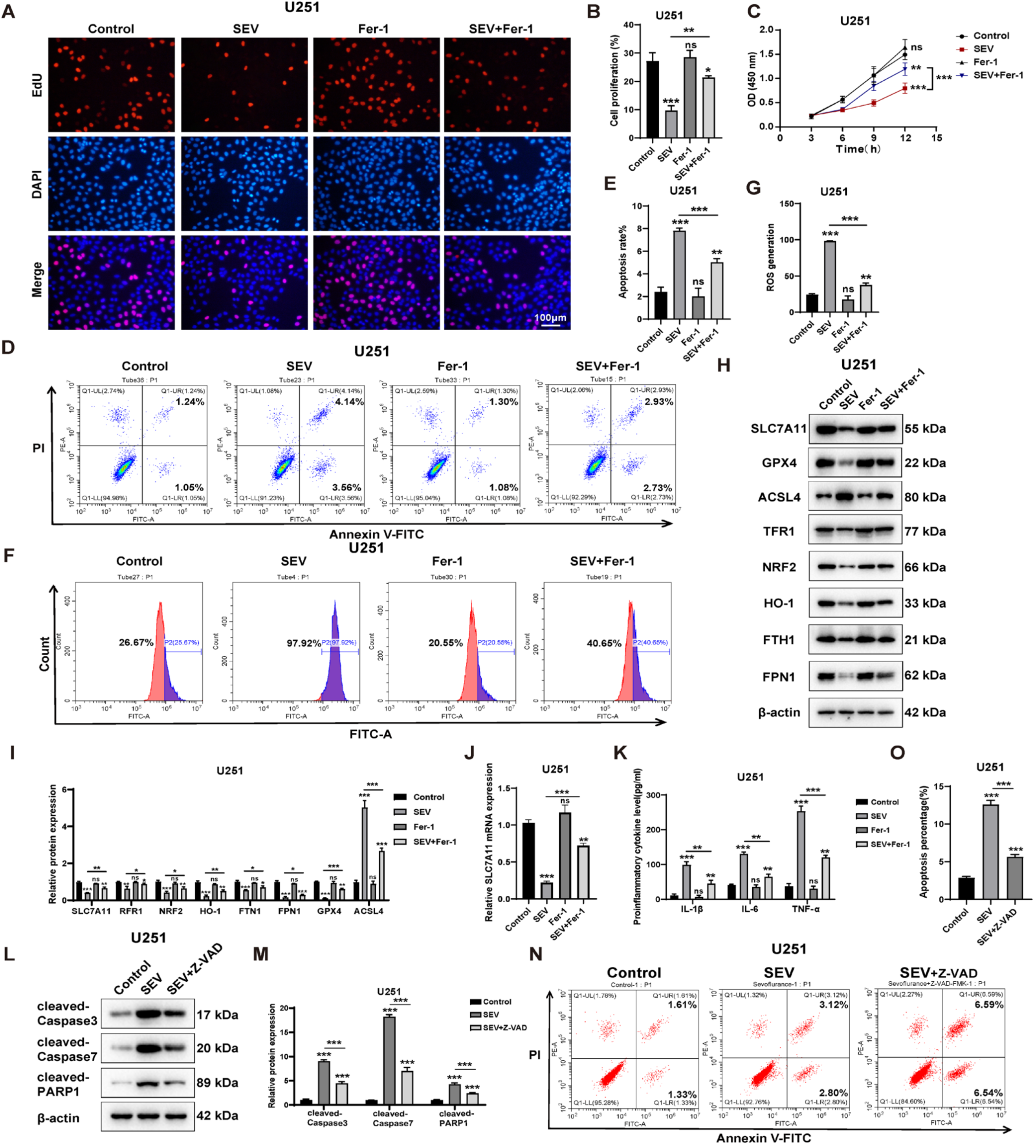
Ferrostatin‐1 improved sevoflurane‐induced downregulation of SLC7A11 expression leading to ferroptosis in astrocytes. (A) EdU assay to detect cell proliferation capacity. (B) Statistical analysis of (A). (C) CCK‐8 assay to detect cell viability. (D) Flow cytometry to detect apoptosis. (E) Statistical analysis of (D). (F) Flow cytometry to detect ROS. (G) Statistical analysis of (F). (H) Protein expression levels of SLC7A11, GPX4, ACSL4, FPN1, TFR1, FTH1, NRF2 and HO‐1 in U251 astrocyte. (I) Statistical analysis of (H). (J) mRNA expression levels of SLC7A11. (K) Inflammation factor levels in medium. (L) Protein expression levels of cleaved‐caspase3, cleaved‐caspase7 and cleaved‐PARP1 in U251 astrocyte. (M) Statistical analysis of (H). (N) Flow cytometry to detect apoptosis. (O) Statistical analysis of (F). Scar bar = 100 μm. *n* = 3 biologically independent samples.'ns' *p* ≥ 0.05; *p < 0.05; **p < 0.01; ***p < 0.001.

We treated U251 cells with 0% and 3.6% sevoflurane, followed by treatment with 2 μM of the ferroptosis inhibitor ferrostatin‐1, using the 0% sevoflurane condition as a control group. Initially, we assessed cell proliferation through EdU and CCK8 staining experiments and found that the intervention with ferrostatin‐1 significantly rescued the suppressive effect of sevoflurane on cell proliferation (Figure 7A–C). Further validation of ferrostatin‐1's rescue effect was obtained through flow cytometry analysis of cell apoptosis (Figure 7D,E). Subsequently, we verified whether sevoflurane induced ferroptosis through ferrostatin‐1 intervention. By measuring intracellular ROS levels using DCFH‐DA, we observed that the extensive ROS generated by sevoflurane was significantly inhibited due to the intervention of ferrostatin‐1 (Figure 7F,G). In addition, compared to the group treated with sevoflurane alone, the group treated with both sevoflurane and ferrostatin‐1 showed a significant increase in the expression levels of key ferroptosis regulatory proteins SLC7A11, GPX4, TFR1, NRF2, HO‐1, FTH1 and FPN1, along with a notable suppression of ACSL4 expression (Figure 7H,I). This further suggests that ferrostatin‐1 can inhibit sevoflurane‐induced ferroptosis in astrocytes. We further examined the mRNA expression levels of SLC7A11, which similarly conformed to the trends observed in its protein expression (Figure 7J). Additionally, through ELISA assays of the cell culture medium, we discovered that ferrostatin‐1 treatment also significantly inhibited the secretion of inflammatory factors (Figure 7K).

Furthermore, since we observed in Figure 6K that sevoflurane can induce apoptosis in addition to ferroptosis, we examined its effects on apoptosis‐related markers, cleaved‐caspase3, cleaved‐caspase7 and cleaved‐PARP. We found that sevoflurane treatment significantly upregulated their expression, while the pan‐caspase inhibitor Z‐VAD‐FMK rescued this phenomenon (Figure 7L,M). Further Annexin V/PI double staining also indicated that Z‐VAD‐FMK could significantly rescue sevoflurane‐induced apoptosis in astrocytes (Figure 7N,O).

## 4 Discussion

Neurological diseases remain the main killer worldwide [19, 20, 21, 22, 23]. Recent research elucidates the complex interactions among anaesthetics, astrocytes and cerebral damage, revealing a complex landscape where astrocytes are crucial in modulating the impact of anaesthetics, thereby playing a significant role in both the exacerbation and mitigation of cerebral damage. For instance, Liu et al. [24] elucidated how parabrachial nucleus astrocytes regulate wakefulness and anaesthesia recovery, indicating that astrocyte activation or inhibition directly impacts isoflurane sensitivity and anaesthesia emergence time. Similarly, Fu et al. [25] demonstrated that isoflurane disrupts olfactory function by altering neuronal activities within the olfactory bulb, with astrocyte activation playing a significant role. Furthermore, interventions targeting astrocytes have shown promise in mitigating cognitive dysfunction postanaesthesia, as seen in studies where electroacupuncture was used to influence astrocyte activity and improve outcomes [26]. These findings underscore the potential of targeting astrocyte functions to develop therapeutic strategies for anaesthesia‐induced cognitive impairment and brain injury, offering a new avenue for enhancing patient recovery and minimising neurological complications associated with surgical procedures.

Advancements in understanding the intricacies of brain injury, ferroptosis and the role of astrocytes have unveiled promising therapeutic targets. For example, studies have demonstrated the selective killing effect of dihydroartemisinin on glioblastoma via GPX4 inhibition, suggesting a novel treatment direction [27]. Furthermore, the involvement of ferroptosis in conditions like Alzheimer's disease has been highlighted, with nicotinamide adenine dinucleotide phosphate oxidase 4 (NOX4) contributing to the ferroptosis of astrocytes through the induction of lipid peroxidation mediated by oxidative stress [28]. The discovery of astrocytes' pivotal role in exacerbating α‐synuclein pathology via the C3‐C3aR signal pathway in Parkinson disease underscores the potential for targeting glial cells in neurodegenerative disease treatment [29]. Additionally, therapeutic applications of hinokitiol may offer potential benefits in alleviating neuronal ferroptosis through the activation of the Keap1/NRF2/HO‐1 signalling pathway in cases of traumatic brain injury [30]. These findings underscore the complexity of brain injury mechanisms and the potential for innovative treatments targeting ferroptosis and astrocyte function, opening new avenues for addressing neurodegenerative diseases and brain injuries.

Our study explored the effects of sevoflurane treatment on the offspring of pregnant mice and on the human astrocytoma cell line U251, focusing on the regulatory role of SLC7A11 in astrocyte ferroptosis and its interaction with sevoflurane. The results indicate that sevoflurane treatment led to learning and memory deficits, reduced exploratory behaviour and increased anxiety towards new environments in the offspring, along with brain tissue structural abnormalities, which were associated with reduction in the levels of the ferroptosis suppressor, SLC7A11. In U251 astrocytoma cells, sevoflurane treatment caused a reduction in cell viability, upregulation of inflammatory factors, a rise in ROS concentrations and changes in the expression of SLC7A11 and related ferroptosis regulatory factors (GPX4, ACSL4, FPN1, TFR1, FTH1, NRF2 and HO‐1), further supporting the hypothesis that sevoflurane induces astrocyte ferroptosis through the regulation of SLC7A11. Rescue experiments with FERROSTATIN‐1 confirmed the key role of ferroptosis in sevoflurane‐induced neurotoxicity, providing a mechanistic explanation for the neural damage induced by sevoflurane through the downregulation of SLC7A11 and the activation of ferroptosis. On the other hand, we also observed that in addition to ferroptosis, sevoflurane induced apoptosis in astrocytes. This finding suggests that sevoflurane‐induced neurotoxicity is not mediated through a single pathway but may involve multiple mechanisms of cell death acting together. Additionally, molecular docking experiments suggest that sevoflurane may directly interact with SLC7A11, affecting its function.

In recent research, the intricate interplay among SLC7A11, astrocytes and ferroptosis has garnered significant attention in the field of neurobiology. Dai et al. and Liu et al. [31, 32] highlighted the protective role of SLC7A11 overexpression in mitigating ferroptosis and hypoxic–ischaemic brain damage, suggesting a potential therapeutic target for brain injuries. Astrocytes, which are essential for neuronal vitality, have been shown to influence ferroptosis via mechanisms that include SLC7A11. Research by Liang et al. has highlighted that neurotoxic A1 astrocytes can induce neuronal ferroptosis in epilepsy, illustrating the intricate function of astrocytes in neurological disorders [33]. Furthermore, studies by Li et al. [34] and Zheng et al. [35] elucidate the mechanisms through which ferroptosis contributes to the aetiology of multiple neurodegenerative conditions, such as Parkinson disease and hypoxic–ischaemic brain damage, thus offering new avenues for therapeutic interventions. The results of this study highlight the pivotal functions of SLC7A11 and astrocytes in modulating ferroptosis, paving the way for novel strategies in the treatment of neurological diseases.

Our findings extend these observations by demonstrating the direct consequences of sevoflurane treatment on learning, memory and behavioural outcomes in mice, alongside molecular alterations in cell models, thus bridging the gap between cellular mechanisms and behavioural phenotypes. Our study notably advances the field by integrating behavioural outcomes with molecular mechanisms, providing a comprehensive view of sevoflurane's neurotoxicity. Furthermore, the utilisation of molecular docking to suggest a direct interaction between sevoflurane and SLC7A11 is a novel approach that could inspire future investigations into the molecular targets of anaesthetic agents and their impact on ferroptosis. Looking ahead, further research could explore alternative anaesthetic agents and their comparative effects on SLC7A11 expression and ferroptosis in astrocytes. Additionally, investigating the potential for pharmacological intervention with ferroptosis inhibitors in the context of anaesthesia‐induced neurotoxicity could offer new therapeutic strategies. Investigating the enduring impacts of sevoflurane exposure on neurodevelopment and cognitive abilities using a broader range of animal models may yield more comprehensive evaluations of its safety characteristics. Moreover, exploring the genetic manipulation of SLC7A11 and other ferroptosis‐related genes in specific neural cell types could unravel cell‐type–specific roles in brain health and disease, paving the way for targeted therapeutic approaches. Overall, this study reveals a new mechanism of sevoflurane‐induced astrocyte damage through the regulation of SLC7A11 and related ferroptosis pathways, offering a new perspective for assessing the neurotoxicity and safety of anaesthetic drugs.

## 5 Limitation

However, several limitations and areas for improvement exist. The sample sizes for both scRNA‐seq and bulk RNA‐seq datasets are relatively small, limiting the generalisability of the findings. The study focuses on early‐stage responses, but the long‐term effects of sevoflurane on neurodevelopment and behaviour are not fully explored. Longitudinal studies are needed to understand chronic impacts. The exact molecular mechanisms by which sevoflurane regulates Slc7a11 expression remain unclear, necessitating further research on epigenetic modifications, transcription factors or signalling pathways involved. The study observed neuroinflammation but did not thoroughly investigate interactions between brain cell types.

Behavioural assays indicated neurocognitive deficits, but underlying neural circuitry and synaptic changes were not examined. Future research should use techniques like electrophysiology and optogenetics. Other forms of cell death (e.g., apoptosis and necroptosis) and their contributions to neurotoxicity need comprehensive analysis. Translational aspects were not fully explored. Investigating ferroptosis inhibitors in vivo and their therapeutic potential, along with safer alternative anaesthetics, would be crucial for clinical application. Addressing these limitations and focusing on interactions, neural circuitry changes and translational applications will enhance understanding and treatment of anaesthesia‐induced neurotoxicity.

In summary, while this study provides important insights into the role of Slc7a11 in sevoflurane‐induced neurotoxicity, addressing the limitations of sample size, in vivo validation, long‐term effects and mechanistic understanding will be crucial in future research. Further investigations into the interactions among different cell types, neural circuitry changes and translational applications will enhance our understanding and potential therapeutic strategies for anaesthesia‐induced neurotoxicity.

## 6 Conclusion

This study provides mechanistic insights into sevoflurane‐induced neurotoxicity, emphasising the importance of SLC7A11 in regulating astrocyte ferroptosis. Our findings highlight the potential for targeting ferroptosis pathways to mitigate the adverse effects of sevoflurane anaesthesia.

## Author Contributions

**Xiaolan Hu:** data curation (equal), formal analysis (equal), investigation (equal), methodology (equal), writing – original draft (equal). **Yiping Zhang:** data curation (equal), formal analysis (equal), investigation (equal), methodology (equal), writing – original draft (equal). **Lian Guo:** methodology (lead), software (lead), validation (equal). **Renjie Xiao:** methodology (supporting), software (supporting), validation (equal). **Linhui Yuan:** conceptualization (supporting), funding acquisition (lead), project administration (supporting), resources (equal), supervision (lead), visualization (equal), writing – review and editing (equal). **Fen Liu:** conceptualization (lead), investigation (equal), project administration (equal), visualization (equal), writing – review and editing (equal).

## Ethics Statement

All animal experiments were conducted according to guidelines approved by the Ethics Committee for Laboratory Animal Welfare of the First Affiliated Hospital of Nanchang University (Approval Number: CDYFY‐IACUC‐202307QR005).

## Conflicts of Interest

The authors declare no conflicts of interest.

## Supporting information


**Figure S1.** Gene function and pathway enrichment analysis of DEGs.

**Table S1.** Primer sequence.
**Table S2.** Proportions of distinct cell types in control and sevoflurane‐treated cortex samples.

## Data Availability

The sequencing datasets used in this study can be found in the GEO database under accession numbers GSE139012 and GSE155770. Experimental data supporting the findings of this study are available from the corresponding author upon reasonable request.
