# Supplementary figures and images for: Comprehensive Analysis of Bulk RNA‐Seq and Single‐Cell RNA‐Seq Data Unveils Sevoflurane‐Induced Neurotoxicity Through SLC7A11‐Associated Ferroptosis
Source: J Cell Mol Med. 2024 Dec 26;28(24):e70307. doi: 10.1111/jcmm.70307 (PMC11670868; doi:10.1111/jcmm.70307)

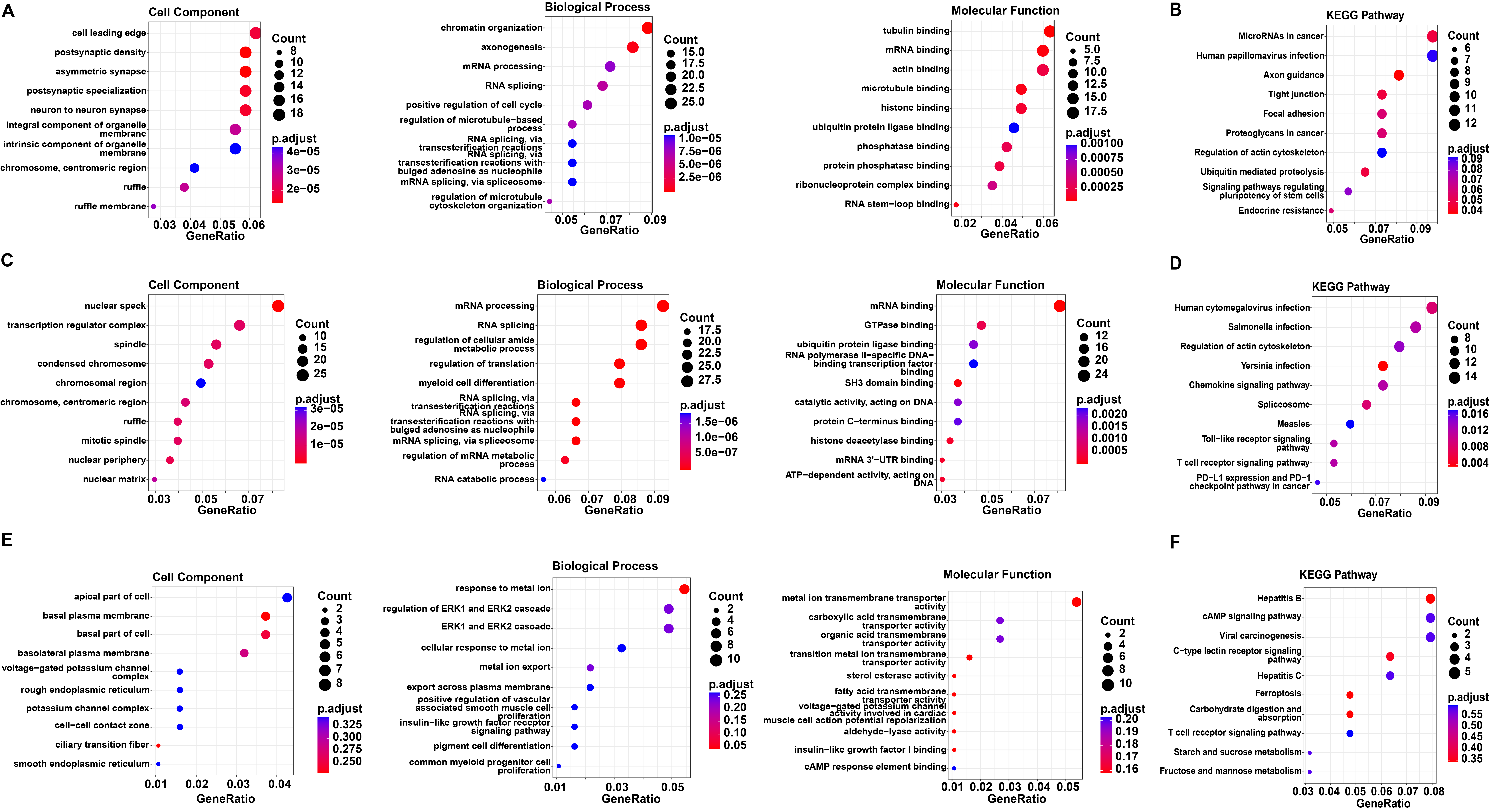

Supplement: Supplementary file 1 — Figure S1. Gene function and pathway enrichment analysis of DEGs. [file JCMM-28-e70307-s002.png]
