# Supplementary material for: Comprehensive Analysis of Bulk RNA‐Seq and Single‐Cell RNA‐Seq Data Unveils Sevoflurane‐Induced Neurotoxicity Through SLC7A11‐Associated Ferroptosis
Source: J Cell Mol Med. 2024 Dec 26;28(24):e70307. doi: 10.1111/jcmm.70307 (PMC11670868; doi:10.1111/jcmm.70307)
Supplement: Supplementary file 2 — Table S1. Primer sequence. Table S2. Proportions of distinct cell types in control and sevoflurane‐treated cortex samples. [file JCMM-28-e70307-s001.docx]

**Supplementary tables**

**Table S1. Primer sequence.**

| SLC7A11 | forward | 5'-AGGGCATACTCCAGAACACG-3′ |
| --- | --- | --- |
|  | reverse | 5'-GGACCAAAGACCTCCAGAATG-3′ |
| β-actin | forward | 5'-GTCCCTCACCCTCCCAAAAG-3′ |
|  | reverse | 5'-GCTGCCTCAACACCTCAACCC-3′ |

**Table S2.** **Proportions of distinct cell types in control and sevoflurane-treated cortex samples.**

| **Cell types** | **Control** | **Sevoflurane** |
| --- | --- | --- |
| Astrocytes | 3332 (16.26%) | 2998 (13.21%) |
| Endothelial cells | 1131 (5.52%) | 1175 (5.18%) |
| Ependymal | 972 (4.74%) | 936 (4.13%) |
| Epithelial cells | 3039 (14.83%) | 3478 (15.33%) |
| Fibroblasts activated | 319 (1.56%) | 251 (1.11%) |
| Fibroblasts senescent | 841 (4.11%) | 1385 (6.1%) |
| Macrophages | 1454 (7.1%) | 1706 (7.52%) |
| Microglia | 1313 (6.41%) | 1464 (6.45%) |
| Neurons | 362 (1.77%) | 417 (1.84%) |
| NPC_S1 | 2060 (10.06%) | 3072 (13.54%) |
| NPC_S2 | 2859 (13.96%) | 2915 (12.85%) |
| Oligodendrocytes | 623 (3.04%) | 545 (2.4%) |
| Tgd | 2182 (10.65%) | 2347 (10.34%) |
